# Introductory Overview: Anticipating and Preparing for Emerging Skills and Jobs—Issues, Concerns, and Prospects

**DOI:** 10.1007/978-981-15-7018-6_1

**Published:** 2020-11-03

**Authors:** Brajesh Panth, Rupert Maclean

**Affiliations:** 20 Education Sector Group, Asian Development Bank, Metro Manila, Philippines; 21 School of Education, RMIT University, Melbourne, VIC Australia; 22 Education Sector Group, Asian Development Bank, Manila, Philippines; 23 Royal Melbourne Institute of Technology (RMIT) University, Melbourne, Australia

## Abstract

Education provides the foundation for skills and lifelong learning opportunities to thrive in professional and social life. However, unlike in the past, the current generation of learners is facing unprecedented uncertainty in how they anticipate and prepare for emerging skills and jobs, due to the impact of automation and continuous technological disruptions.

Education provides the foundation for skills and lifelong learning opportunities to thrive in professional and social life. However, unlike in the past, the current generation of learners is facing unprecedented uncertainty in how they anticipate and prepare for emerging skills and jobs, due to the impact of automation and continuous technological disruptions. On the one hand, many countries are facing a “ learning crisis” although they have achieved remarkable progress in improving access to education at all levels. On the other hand, skills mismatches are growing to a point where many graduates are unable to get jobs, while employers are often unable to fill vacancies due to the changing nature of skills and jobs. The current schooling systems, which was founded around 100–150 years ago to enhance the efficiency of the first and second industrial revolutions, is no longer adequate for people to thrive and prosper in the current world, which is increasingly driven by artificial intelligence, automation, and innovation.

Research evidence clearly shows that student learning outcomes, as measured by tests such as the Organization for Economic Cooperation and Development’s (OECD) Programme for International Student Assessment (PISA), and Trends in International Mathematics and Science Study (TIMSS), are more closely associated with economic development and innovation than are mean years of schooling (Hanushek and Woessmann 2012).^1^ Most developing countries have made remarkable progress in enhancing access to education, including an improved gender balance at all levels of education. However, it is also clear from research that going to schools does not guarantee learning (World Bank 2018).^2^ Research on the outcomes of the Programme for the International Assessment of Adult Competencies (PIAAC) also shows that poor learning at the initial stage can continue at the higher levels (Pritchett 2019).^3^ Therefore, without ensuring learning for all, life choices of students who are disproportionately affected by poor learning, will be more limited.

It is critical to understand that the same strategy that has worked well to expand access to primary and secondary education, may not contribute to making substantial improvements in student learning outcomes (Pritchett 2019).^4^ The current education system is no doubt overdue for essential transformation to adapt to the needs of the Fourth Industrial Revolution (4IR), which is described as a stage of continuous digital disruptions, involving the fast-changing nature of jobs and short shelf life of skills. In order to prepare self-directed learners who can reskill and upskill themselves, throughout their long professional life, to adapt to new and emerging requirements, it is important to equip learners with different types of skills (whether they be cognitive, noncognitive, and/or occupational skills) .^5^ This means it is essential to rethink and reimagine teaching and learning, since learning is not only happening in formal settings such as in schools, colleges, and training centers, but also in the home, workplace, and in other nonformal and informal settings.

At the postsecondary level, there is a growing advocacy for competency-based and performance-based education away from prestige based, elitist education, particularly in the United States, in order to enhance employability and reduce the costs of tertiary education.^6^ This is also happening in a wide range of other countries. It has been estimated that about 80% of all jobs worldwide require some form of vocational skills (Maclean and Wilson 2009). Vocational skills are classified as those which allow an individual to master a particular subject, procedure, or area of understanding, that is applicable to a work career. With the acquisition of knowledge, skills, and values for working, there is an increase in opportunities for productive work and sustainable livelihoods. According to UNESCO, “ TVET refers to those aspects of the education process that involves, in addition to solid general education, the study of technologies and related sciences, and the acquisition of practical skills, attitudes, understanding, and knowledge related to work and occupations in various sectors of economic life”. This is sometimes also called Applied Learning.

The urgent challenge facing all countries is to take effective action to match their systems of education and training to meet the actual, and rapidly evolving, employment needs of their economy and society’s vocational skills. That is why relevant and high-quality basic skills for all comprising foundational skills is vitally important for the continuing prosperity and development of all countries, and to help achieve greater equity, justice, and fairness in the countries concerned.

This book is concerned with taking a holistic approach to different types of skills that employers are looking for and examining how education and training systems are responding to rapidly changing requirements in light of the 4IR. The book highlights the importance of different types of skills (cognitive, noncognitive, and occupational), as well as lifelong learning, to improve the life choices of a growing number of mobile workforces that expect to have productive employment during an increasingly longer working life and with a changing definition of jobs.

There are several important matters that greatly impact on education and training during what is a period of unprecedented change due to disruptions in virtually all areas. The shift from the industrial age to the information age and knowledge economies, and from localization toward globalization, has considerable implications for TVET, as do moves to effectively harness information and communications technologies.

**Dominant importance of the 4IR**: As Klaus Schwab, Founder of the World Economic Forum has pointed out: “we stand on the brink of a technological revolution that will fundamentally alter the way in which we live, work and relate to one another” (Tadeu and Brigas 2018). With the 4IR there have been major innovations in production techniques and in the ways of organizing work, accommodating changing employment arrangements, and increasing the use of technology, in particular high-speed internet, robotics, and artificial intelligence. There has also been a shift toward more and more occupations requiring an expanding repertoire of high-level skills and technology.

 The 4IR has profound implications for how we best prepare people for the changing world of work, particularly in the area of applied learning and skills development for employability and for life. For example, it is estimated that 75% of future jobs will involve science, technology, engineering, and mathematics (STEM) knowledge and skills (Maclean 2019). But it is also important to integrate and promote the “arts” to stimulate imagination, creativity, and entrepreneurship.

The provision of such higher-order cognitive skills cannot be adequately satisfied by our present education and training systems. The types of changes occurring will mean that increasing numbers of people will need new skills, reskilling, and upskilling.

**Twenty-first Century Skills and the greening of economies**: In order to combine human intelligence to innovate with the computing power of machines, countries need to prepare their workforce with vitally important twenty-first century skills such as critical thinking, creativity, communication, collaboration, problem-solving, cross-cultural competencies, work ethic, empathy and social, emotional and digital intelligences. In other words, there is a need for workers with “ multiple intelligence”. Such skills constitute a different dimension of teaching and learning and, alongside core literacy and numeracy skills, are viewed as important for helping students ensure a successful transition to life and work after education, and to remain up-to-date. It is also equally important to foster academic and sustainable mindsets so that educators are able to improve students’ research and development capacity for sustainable development, collaboration, and positive attitudes toward healthy and green growth.

Referring to anticipating and preparing for emerging skills and jobs, Satya Nadella, the CEO of the technology giant Microsoft, says that society needs to acknowledge the massive displacement of jobs that has occurred, and will increasingly occur, due to globalization. He said that “What has happened in the first phase of globalization is that, a lot was created. A middle class was created even in Asia. The second phase of globalization has to tackle the inequities that got created in every country. I see a future of innovation which distributes computing and human collaboration around technology”.

**Quality education and relevant skills to catalyze economic and social transformation**: Many reports are highlighting the transformational changes affecting the ways we work and live. The nature of work and the structure of economies are being changed through rapid changes in technology, unprecedented labor mobility, globalization, and demographic changes. The evolving world of work calls into question the skills that people need to develop in order to navigate these changes. Those who are able to adapt to emerging needs in a flexible and integrated manner are more likely to thrive. What this means is the need to transform education and training systems to mimic the real-world situation through experiential and project-based learning. In this regard, there are four key questions that need to be answered: (1) what are employers looking for when recruiting for their workplaces? (2) what are individuals looking for in their life’s work? (3) what role does education and training play in addressing these expectations? and (4) how can technology enhance learning and employability?

**Equity issues with particular reference to gender and youth**: There are more than 100 million school-age children and youth in the Asia and Pacific region, mostly girls/women and disadvantaged groups, that are either out of school or lack employable skills despite schooling and college education, due to the poor quality of education received. To realize the ambitious quantitative and qualitative targets of Sustainable Development Goal 4 (on education) and Goal 8 (on employment), it is important to prioritize the following three critical areas: (i) going beyond physical access, to enhancing opportunities for females, disadvantaged groups and people with disabilities; (ii) finding nontraditional ways of preparing such target groups; and (iii) promoting partnerships to enhance the delivery and quality of education and training through better targeting.

**Need to be future-oriented**: It is widely recognized that the rapid pace of technological, social, demographic, and political changes will continue to accelerate and create more volatility and uncertainty in the future. But this will also create new opportunities, as new jobs requiring new set of skills are being created. There is a need to develop a future-oriented curriculum framework for education and training so that learners can prepare and adapt to emerging needs with confidence and know how to reskill and upskill to remain current and effective.

**Vocationalization of education**: Many countries view secondary education as academic preparation for entrance to higher (postsecondary) education. Over the past two decades, greater attention has been given to the relevance of what is taught at the secondary level to best prepare learners for the workforce and vocational work. In doing so the aim has been to meet the needs of students who not only go onto higher education, but also those likely to enter the workforce directly from secondary school. This trend has resulted in what has been called the “vocationalization of secondary education”. Vocationalized secondary education refers to a curriculum which remains overwhelmingly “general” with foundational skills (cognitive, digital, and soft skills), but which also includes vocational subjects as a minor portion. In addition, some tertiary institutions, including famous, high standard institutions such as the University of Oxford in the United Kingdom, are also exploring the “vocationalization of higher education” to prepare their graduates for a smooth transition into the workforce. This is a “ competency-based” approach.^7^

## Assessing the Appropriateness of Various Skills for Employability

When examining the matter of “anticipating and preparing for emerging skills and jobs”, there are several key matters that need to be addressed regarding the appropriateness of the repertoire of skills necessary for effective employability.

- The definition of foundational skills is changing from the 3Rs (reading, writing, arithmetic) which have traditionally been addressed in all education and training programs, to the 3Rs plus digital literacy, soft skills, and occupational skills. A major challenge is how to both teach and assess the soft skills over time.
- Many developing countries are facing a “ learning crisis”. The reality is that despite significant improvements in enrollment and attendance, many students are not successfully learning in schools due to a wide variety of reasons such as an irrelevant and outdated curriculum, unprepared teachers, and a weak or incomplete assessment system. In many cases, teachers are not able to teach students at the grade level due to diverse needs and an uneven readiness of students, and weak assessment. As a result, fast learners are progressing well while slow learners lag behind or they end up preparing for the tests through rote learning or dropout.
- Many students going to TVET programs come from weak schooling, and lack the foundational skills needed to better understand and apply what they learn. It is important to ensure that TVET programs also include foundational skills that help learners to learn better and create opportunities for them to pursue different pathways by investing in lifelong learning and strengthening their skills as a continuous process. Employers also need to be incentivized to invest in reskilling and upskilling their workforce to be competitive, productive, and innovative.
- Online blended and simulated learning is evolving as a cost-effective way of massifying teaching and learning without compromising on education quality. For instance, Indonesia is embarking on a large scale online learning ecosystem to expand enrollment in regular higher education by allowing students in universities to earn up to 50% credit from online courses. Such initiatives also help to strengthen lifelong learning. Curriculum could become highly relevant by curating and including some of the best online courses offered by the providers of massive open online courses (MOOCs). Countries could draw on world-class courses to prepare teachers and to ramp up skills in priority areas, such as machine learning, artificial intelligence, and big data analytics. With continued improvements in technology, it will be possible to make learning more authentic and experiential. However, it is important to collaborate with proven regional and global partners to ensure high-quality blended learning.
- The demand for online learning has surged during the COVID-19 pandemic due to school closures in over 180 countries, affecting over 1.5 billion students worldwide. Governments are scrambling to provide online learning to students to continue uninterrupted learning. Public education has never seen such a large scale of online learning initiative. Some important lessons have come out from the People’s Republic of China^8^: (i) more equitable access to infrastructure (connectivity, platforms, and devices), (ii) preparing teachers to manage and deliver high-quality instructions, (iii) ensuring high-quality content aligned to national curriculum and reliable assessment tools, (iv) preparing students to learn at their own pace, (v) ensuring parents are able to get feedback on their children’s performance to support learning, and (vi) facilitating partnership between public and private institutions to ensure synergy in enhancing teaching and learning. It is important to draw on other good practices to build on the experience emanating from the COVID-19 pandemic.
- Artificial Intelligence and big data analytics are very promising in a number of areas, such as (i) to help teachers assess students continuously through personalized and adaptive learning to ensure that everyone is acquiring the needed competencies; (ii) to develop a real-time labor market intelligence system to identify how occupations are changing, including how new ones are emerging and old ones are disappearing, leading to reduced skills mismatches and improved matching between emerging needs and individual profiles of job seekers; and (iii) to make teaching and learning more transparent and accountable by generating and sharing data on learning and engagement.

## Overview of the Contents of This Book

This edited volume on *Anticipating and Preparing for Emerging Skills and Jobs* consists of a compilation of eight parts, including the introductory and concluding parts, and 40 articles which refers to three levels of education (K-12, TVET, and higher education) as well as important themes such as educational technology, and technology platforms for bridging skills gaps and mismatches, and cross-sectoral collaboration for skills development.

The book draws on presentations made at the past four International Skills Forums organized by ADB in its headquarters in Manila. The articles presented here seek to capture the essence of the important topics discussed at these ADB Forums: current priorities on TVET, innovative practices in skills development, anticipating and preparing for emerging skills and jobs, and, the future of skills and jobs in the age of digital disruption.

The 40 articles included here are clustered under major, uniting themes concerning key aspects of education and training for the changing world of work. Each article is intended to be a discussion starter for the particular matter examined.

These articles have been written by eminent researchers, policy-makers, and practitioners working in universities, Ministries of Education and Ministries of Labor, international education for development agencies such as the OECD and ADB, and various other nongovernment organizations. Although the authors have been drawn worldwide there is an emphasis on the Asia and Pacific region.

The objective of this volume is to

1. Showcase transformational practices to equip youth with employable and life skills
2. Highlight the perspectives of those from government, universities, the private sector, international development agencies, and other partners involved in education and training to showcase innovative good practices
3. Demonstrate and highlight real examples of effectively transforming education and training with technology, eLearing, and innovative partnerships
4. Share new ideas at the regional and country levels, and build upon current knowledge and experience in implementing effective and relevant education training systems that are more responsive to change.

Chapters in this book are organized under eight main sections. The introductory overview (Part 1) sets the overall context. It examines the implications of automation on jobs and the need to focus on higher level skills for developing countries to leapfrog. While EdTech solutions, including online learning, are expanding due to technological advancements, the focus needs to be on improving the quality of education for countries to ensure sustained economic growth and building evidence. Universities have to innovate to create academic entrepreneurs by working closely with industry. For people to prosper and countries to sustain development, everyone needs to develop the right skills to take advantage of technological, economic, and social progress.

Kindergarten to Grade 12 (K to 12) reforms are then examined (Part 2) by looking at effective ways of boosting student learning, including as measured by PISA through technical partnership (OECD’s experience on PISA); drawing on locally driven models to develop effective learning strategies in schools by improving student engagement where highly prepared teachers may be scarce (Philippines); using technology-based assessment tools to support teachers to continuously monitor student learning levels and adjust pedagogy accordingly (EdTech solution); partnering with nongovernment providers to expand quality education for all (Pakistan); and drawing lessons from successful developing countries on how they have improved student learning outcomes (Viet Nam).

The book then moves on to examine the specific impact of such transformational changes on TVET (Part 3). Examples presented highlight how the dual training approach is preparing job-ready graduates by addressing skills mismatches (Philippines); how a more strategic focus on infrastructure and pedagogy innovation can transform TVET to raise the image of TVET and prepare job-ready graduates (Singapore); how partnerships with industry associations can lead to quality-assured and responsive TVET programs that are owned and supported by employers (New Zealand); how market-driven TVET, targeting priority sectors, can prepare youth for meaningful jobs (India); how ADB’s support to skills development is working closely with industry associations and proven institutions in priority sectors (Bangladesh); and how TVET programs need to prepare trainers and link with information and communications technology to enhance the quality of training (PRC).

Higher education has an important role to play in promoting higher level skills (Part 4). The articles highlight how world- class universities are being set up to promote innovation and research and development (Hong Kong, China; Indonesia; and the Republic of Korea); how university-industry linkages are promoting research and development and commercialization (Shenzhen, PRC); how ADB’s support to the University of the South Pacific is taking a regional approach to strengthening higher education institutions in the Pacific; and how universities are innovating by helping to establish start-ups and an entrepreneurship ecosystem (Republic of Korea).

 Education Technology (EdTech) has the potential to improve teaching and learning at all levels of education (Part 5). Blended learning can support universal access to high-quality and relevant education and training including teacher professional development at scale. While EdTech has a huge potential to enhance the quality of TVET, concerted efforts are needed to bridge existing skills gaps. The power of mobile technologies is proving to be effective in massifying access to quality education, but this will require large-scale public-private partnerships. MOOCs curated by global experts are also helping learners across the globe to learn new skills linked to global demand. Specialized training for IT industry can be effective in meeting global demand of such skills. Similarly, coding skills can be taught from early age to prepare learners with design thinking.

 Artificial Intelligence and big data analytics are fueling the development of technology platforms (Part 6) to support career counseling and guidance (Philippines), workforce transformation by linking labor market information with skills profiles of job seekers (Singapore), improving labor market intelligence to ascertain which occupations are emerging and which ones are disappearing (ADB’s analysis), and preparing the workforce in response to IR4 needs.

Education and training contributes to all the SDGs. Cross-sectoral collaboration is therefore critical for skills development for employability (Part 7). This section examines how skills development can be embedded in infrastructure projects (Mongolia); how science, technology, engineering, arts, and mathematics (STEAM) can help youth to develop leadership, build confidence, and cultivate employability skills (Thailand); how ADB’s support is leading to increase in female enrollment in nontraditional TVET (Tajikistan); how countries are developing sectoral approaches to skills development in energy, automobile, textile, and IT (European Training Foundation’s Torino process); and how lifelong learning can stimulate demand for skilling and upskilling (Singapore).

The final section (Part 8) draws together all the ideas and case study material presented in earlier sections of the book by providing specific, concrete conclusions and recommendations regarding the most effective ways to move ahead to anticipate and prepare for emerging skills and jobs.

## Footnotes

1 Hanushek and Woessmann. 2012. Do better schools lead to more growth? Cognitive skills, economic outcomes, and causation. Journal of Economic Growth.

2 World Bank. 2018. World Development Report: Learning to Realize Education’s Promise.

3 Lant Pritchett. February 13, 2019. Research on Improving Systems of Education (RISE). https://www.riseprogramme.org/blog/learning_crisis_at_top.

4 Discussions with Lant Pritchett at the Asian Development Bank. August 2019.

5 Cognitive skills refer to literacy, numeracy, and analytical skills; noncognitive skills refer to soft skills such as critical thinking, creativity, communication, and collaboration; and occupational skills refer to skills needed to perform specific jobs.

6 Horn and Dunagan. June 2018. Innovation and Quality Assurance in Higher Education. Christensen Institute.

7 UNESCO-UNEVOC in Bonn organized a regional UNESCO Forum on “*Vocationalization of Higher Education**: Issues and Prospects*” at the University of Oxford in 2014, with the Education Department in the University of Oxford as partner in organizing the Forum, which addressed the matter of the “Vocationalization of Higher education”.

8 Xu et al. (2020).
